# Exploring Differences in Dietary Diversity and Micronutrient Adequacy between Costa Rican and Mexican Adolescents

**DOI:** 10.3390/children11010064

**Published:** 2024-01-03

**Authors:** Rafael Monge-Rojas, Rulamán Vargas-Quesada, Joaquín Alejandro Marrón-Ponce, Tania G. Sánchez-Pimienta, Carolina Batis, Sonia Rodríguez-Ramírez

**Affiliations:** 1 Nutrition and Health Unit, Costa Rican Institute for Research and Education on Nutrition and Health (INCIENSA), Ministry of Health, Tres Ríos 4-2250, Costa Rica; rvargas@inciensa.sa.cr; 2 Nutrition and Health Research Center (CINyS), National Institute of Public Health, Mexico (INSP), Cuernavaca 62100, Mexico; cinys33@insp.mx (J.A.M.-P.); carolina.batis@insp.mx (C.B.); scrodrig@insp.mx (S.R.-R.); 3 CONAHCYT, Nutrition and Health Research Center (CINyS), National Institute of Public Health, Mexico (INSP), Cuernavaca 62100, Mexico

**Keywords:** minimum dietary diversity, micronutrient probability of adequacy, adolescents, Latin American countries

## Abstract

Diet diversity becomes especially relevant during adolescence to satisfy the adequate micronutrient intake. Diet diversity (DD) and micronutrient probability of adequacy (PA) were studied in 818 Costa Rican (CR) and 1202 Mexican (MX) adolescents aged 13–18 years. DD was compared using the Minimum Dietary Diversity (MDD) score. Receiver-operating characteristic (ROC) curves were employed to identify the optimal MDD for each sample from the respective countries. The mean MDD for the overall CR sample was 4.17 ± 1.43 points, and for the MX sample, the mean MDD was 4.68 ± 1.48 points. The proportion of adolescents with a DD was significantly higher in Costa Rica than in Mexico (66.5% vs. 55.6%; *p* < 0.0001). Also, DD was higher in rural Costa Rican adolescents, while no difference was found in the MX adolescents by area of residence. CR adolescents reported significantly higher PA than MX participants for 6 of the 11 micronutrients assessed. The calcium PA in MX adolescents was significantly higher than in the CR sample (MX: 0.84 vs. CR: 0.03; *p* < 0.0001), while low PA was obtained for iron in both countries (CR: 0.01 vs. MX: 0.07; *p* < 0.0001). In Costa Rica and Mexico, nutritional interventions and assessing the compliance of food-fortifying programs are needed to improve the PA of diverse micronutrients.

## 1. Introduction

A diverse diet becomes especially relevant during adolescence since, at this stage of life, nutrient requirements are notably high due to physical growth and biological maturation [1]. Various studies have shown that adolescents living in low- and middle-income countries have low dietary diversity and poor consumption of micronutrient-dense foods, leading to a high prevalence of inadequate intakes of vitamin A, iron, calcium, zinc, and folates [2,3,4,5,6,7], as well as a high prevalence of vitamin B12 deficiency, low serum zinc, and anemia [3].

Dietary variability results from food choice patterns within a population group [8] and comprises traditional foods inherent to its food culture, exemplified by items like corn tortillas and chili peppers in Mexico [9], as well as white rice and beans in Costa Rica [10]. Additionally, it incorporates foods introduced into the culture due to globalization [11].

Several studies indicate that the eating pattern is healthier when it includes diverse food groups [2,12,13,14]. Dietary diversity has long been recognized as a key element of high-quality diets because it is positively correlated with nutrient density and dietary adequacy [12], and because a variety of foods supports better health outcomes [3,12]. Dietary diversity refers to the count of various foods or food groups consumed within a specified reference period [15].

Research on adolescents in Low-Income Countries (LICs) and Middle-Income Countries (MICs) indicates a diversity in Dietary Diversity Scores (DDS), ranging from 3.35 points in India [7] to 6.25 points in South Africa [16]. However, these values lack comparability due to substantial variations in the methodologies used to determine DDS across different studies. To mitigate the extensive variability in studying dietary diversity, it is crucial to adopt a straightforward methodology that aligns with the specific context and food culture of a country or a large geographic area. Addressing this need, the Food and Agriculture Organization (FAO) of the United Nations, along with its partners, introduced the Minimum Dietary Diversity for Women (MDD-W) indicator [17]. This indicator serves as a practical, food-based measure designed to assess dietary diversity and micronutrient adequacy, emphasizing key aspects of diet quality in women of reproductive age.

Dietary diversity scores have been identified as promising measurement tools due to their simplicity of implementation compared to other complex and expensive methods used to monitor micronutrient intake [15]. Although it was validated and intended to be used as a proxy for micronutrient adequacy in women of reproductive age, the Minimum Dietary Diversity for Women (MDD-W) has shown its applicability in other population groups, including adolescents [5,6,18,19]. To simplify the use of the MDD-W, dichotomous versions of it have been proposed. The Food and Agriculture Organization of the United Nations (FAO) outlines that consuming 15 g or more from a minimum of five out of ten defined food groups within a 24 h recall period establishes the minimum dietary diversity (MDD). This level is deemed sufficient to meet the recommended intake of 11 crucial micronutrients, including vitamin A, thiamine, riboflavin, niacin, vitamin B6, folate, vitamin B12, vitamin C, calcium, iron, and zinc [17].

Various studies [5,20,21] have emphasized the importance of assessing the sensitivity and specificity of different MDD cut-off points. This analysis aims to identify the most suitable threshold for avoiding the over-identification of population segments with nutritionally inadequate diets. Based on this principle, other research efforts have established distinct MDD cut-off points, such as ≥4 for Ugandan adolescents [22]. These adjustments aim to minimize measurement errors when estimating the population subgroup at a higher risk of inadequate micronutrient intake. In this context, it was considered relevant to explore differences in dietary diversity and micronutrient adequacy between adolescents from two mestizo-background Latin American countries: Costa Rica and Mexico. This significance is underscored by the fact that Mexican food culture retains a substantial influence from both pre-Columbian traditions and the colonial era [23]. In contrast, Costa Rican food culture exhibits a more pronounced Westernized influence, preserving relatively few elements from both pre-Hispanic culinary traditions and those introduced during Spanish colonization. A notable example of this cultural divergence is evident in the contrasting emphasis on corn culture, which remains a robust food tradition in Mexico, while in Costa Rica, it has been supplanted by the prevalence of wheat culture [24].

The aims of the current study were: (1) to determine the MDD cut-off point for the Costa Rican (CR) and Mexican (MX) adolescents’ diets, (2) to compare dietary diversity between CR and MX adolescents, and (3) to compare the micronutrient probability of adequacy in CR and MX adolescents.

## 2. Materials and Methods

### 2.1. Study Population and Setting

The CR study sample comes from a cross-sectional study conducted in 2017 which included 818 adolescent participants between 13 and 18 years old enrolled in 18 schools (10 in urban areas and 8 in rural areas) in the province of San José, where the largest proportion of adolescents is concentrated [25]. Details of the sample selection have been published elsewhere [6]. The MX study sample included 1202 adolescents between 12 and 19 years and stems from the 2016 Mexican Halfway National Health and Nutrition Survey (ENSANUT MC 2016), which is a probabilistic survey with national, urban, and rural representation conducted by the National Public Health Institute (INSP) of Mexico. Detailed information on the design, sample size, and methodology of the ENSANUT MC 2016 has been described previously [26].

The Bioethics Committee of the Costa Rican Institute for Research and Education in Nutrition and Health (INCIENSA) and the INSP’s Research, Ethics, and Biosafety Committees approved the corresponding study protocols, and all guidelines for human subject research were strictly followed. The Costa Rica study was approved on 5 August 2016, whereas the Mexico study obtained approval on 13 April 2016.

### 2.2. Sociodemographic Variables

Structured questionnaires were used to collect data on sex, age (in years), area of residence, and socioeconomic status (SES) in both countries. In Mexico, localities with fewer than 2500 inhabitants were designated as rural areas, while those with 2500 inhabitants or more were classified as urban [26]. In Costa Rica, urban areas comprise the administrative centers of the country’s cantons, encompassing either part or the entirety of the first district of each canton, as well as centers of other districts and adjacent areas. The determination of urban areas in Costa Rica considers physical and functional criteria, including tangible elements such as quadrants, streets, sidewalks, electricity, and urban services. Conversely, rural areas in Costa Rica encompass populated centers not falling within the aforementioned urban categories. These rural areas exhibit the following characteristics: (1) the land is predominantly used for non-agricultural activities; (2) they consist of 50 or more grouped or contiguous homes, typically spaced no more than 20 m apart; (3) they possess some infrastructure services like home electricity, drinking water, or telephone; and (4) they provide various amenities such as schools, churches, health centers, grocer’s shops, and rural guard stations [27].

In Mexico, socioeconomic status (SES) was determined by employing principal component analysis, considering housing characteristics (roof, wall, and floor materials; drainage; and water availability), as well as possessions such as televisions, computers, radios, etc. This process resulted in a continuous variable, subsequently divided into tertiles representing low, medium, and high socioeconomic levels [26]. In Costa Rica, SES classification utilized information on parental educational levels, ownership of goods, and access to services (e.g., computers, internet, router, cable television, and water heating for the entire house), along with family structure. The k-means procedure was employed for this classification process [28].

### 2.3. Anthropometric Assessment

Adolescents’ height and weight measurements were measured by trained and standardized personnel following the methodology described by Preedy [29], Lothman [30], and Habitch [31]. Body mass index (BMI) values were computed to assess the nutritional status of adolescents, employing the BMI-for-age Z score as per the WHO guidelines [32]. Classifications were established: <−2 denoted underweight, ≥−2 and <+1 represented a healthy weight (eutrophy), ≥+1 and <+2 indicated being overweight, while ≥+2 signified obesity. For data analysis, nutritional status was simplified into two categories: non-overweight (encompassing underweight and eutrophic conditions) and overweight/obesity (including overweight and obese classifications).

### 2.4. Dietary Intake Assessment

Dietary intake information from Costa Rican adolescents (CR) was gathered through 3-day food records (3FR), which participants completed in real time and were subsequently reviewed by nutritionists. To ensure comprehensive coverage, half of the participants were randomly assigned to record their food and beverage intake on Thursday, Friday, and Saturday, while the remaining individuals documented their consumption on Sunday, Monday, and Tuesday. These records were collected over nine months of the school year, spanning from February to November, thereby encompassing seasonal fluctuations in Costa Rica: the rainy season (from May to November) and the dry season (from December to April). This approach aimed to capture both daily and seasonal variations in food consumption patterns. The data processing involved using Epi Info™ software (version 3.5.4, 2008, Atlanta, GA, USA), which utilized information from the School of Human Nutrition database at the University of Costa Rica [33]. Further elaboration on the dietary assessment methodology can be found in a previously published resource [6].

Dietary information regarding Mexican (MX) adolescents was gathered using the 24 h-recall automated multiple-pass method (24HR). Additionally, a subset of adolescents underwent a second 24HR assessment after a two- or three-day interval from the initial one. Both 24HR were conducted on randomly chosen days of the week, encompassing dietary intake reporting from Monday to Sunday. To estimate energy and micronutrient intake, the MX food composition database was utilized [34]. A previously published report has detailed a comprehensive outline of the dietary assessment methodology [26]. The food composition databases utilized in both Mexico and Costa Rica for the analysis of micronutrient consumption data incorporated fortified foods in each respective country.

For comparative purposes between the CR and MX adolescents, only specific days of dietary intake were considered. In the CR dataset, the second day of the 3-day food record was chosen because it represented a weekday for the entire sample, reflecting habitual dietary patterns among adolescents. This choice aimed to prevent bias in the dietary intake analysis, considering the reported differences in food consumption and diet quality between weekdays and weekends among this adolescent cohort [6]. Conversely, in the case of Mexico, the first 24HR assessment was utilized as it encompassed the entire adolescent sample, while the subsequent 24HR assessment covered only a subset of participants.

### 2.5. Minimum Dietary Diversity

The calculation of the MDD followed the Women’s Dietary Diversity Score Project food group classification [17]. Dietary diversity was assessed by assigning a score contingent on the consumption of a minimum of 15 g per day from the 10 food groups defined by the FAO. These groups include starchy staples (grains, roots, and tubers, and plantains), flesh foods (meat, poultry, and fish), dark green leafy vegetables, other vitamin A-rich fruits and vegetables, other vegetables, other fruits, pulses (beans, peas, and lentils), milk and milk products, eggs, and nuts and seeds.

For each food group with an intake of ≥15 g per day and per person, 1 point was allotted, while an intake of <15 g per day received 0 points. This scoring system resulted in a range of 0–10 points, with a higher score indicative of greater dietary diversity. A higher score implies consumption from a broader array of food groups that meet or surpass the 15 g/day threshold. The assessment for MDD was carried out by considering variables such as sex, area of residence, SES, and nutritional status categories, encompassing a comprehensive range of factors for a thorough examination.

### 2.6. Micronutrient Intake Adequacy

The micronutrient intake adequacy was estimated using the probability approach method [35]. Micronutrient intake data obtained from the 3FR (in the CR case) or the two 24HR (in the MX case) were used to determine usual micronutrient intakes. The process involved deriving the best linear unbiased predictors (BLUPs) of usual intake for each micronutrient (including vitamin A, thiamine, riboflavin, niacin, pyridoxine, folate, vitamin B12, vitamin C, zinc, calcium, and iron). To accommodate individual variations in intake, the Software for Intake Distribution Estimation (PC-SIDE) version 1.0 from Iowa State University [36] was employed. Following this, the probability adequacy (PA) was assessed by comparing each BLUP value with its corresponding requirement distribution. This requirement distribution was established using the Estimated Average Requirement (EAR) alongside the standard deviation (EAR multiplied by the coefficient of variance) for each specific micronutrient [37]. The PA was determined utilizing the “normprob” command in Stata, employing the following Equation (1):

(1) PA=normprobBLUPmicronutrient−EARSD

This calculation aimed to ascertain the likelihood of adequacy for each micronutrient based on the contrast between the BLUP and the EAR, normalized by the standard deviation.

The PA for iron was estimated based on an assumed bioavailability of 10%, in accordance with the World Health Organization’s recommendation for diets in developing countries [38]. The PA scale ranged from 0 to 1, indicating the spectrum from not achieving to achieving the recommended intake of the micronutrient. To derive a comprehensive assessment, the PA for each micronutrient across the 11 considered micronutrients was calculated for the adolescents. This individual micronutrient PA was then averaged to obtain the Mean Probability Adequacy (MPA). The MPA represents the average likelihood of meeting the recommended intake across the set of considered micronutrients.

### 2.7. Minimum Dietary Diversity Cut-Off

**Figure 1:**
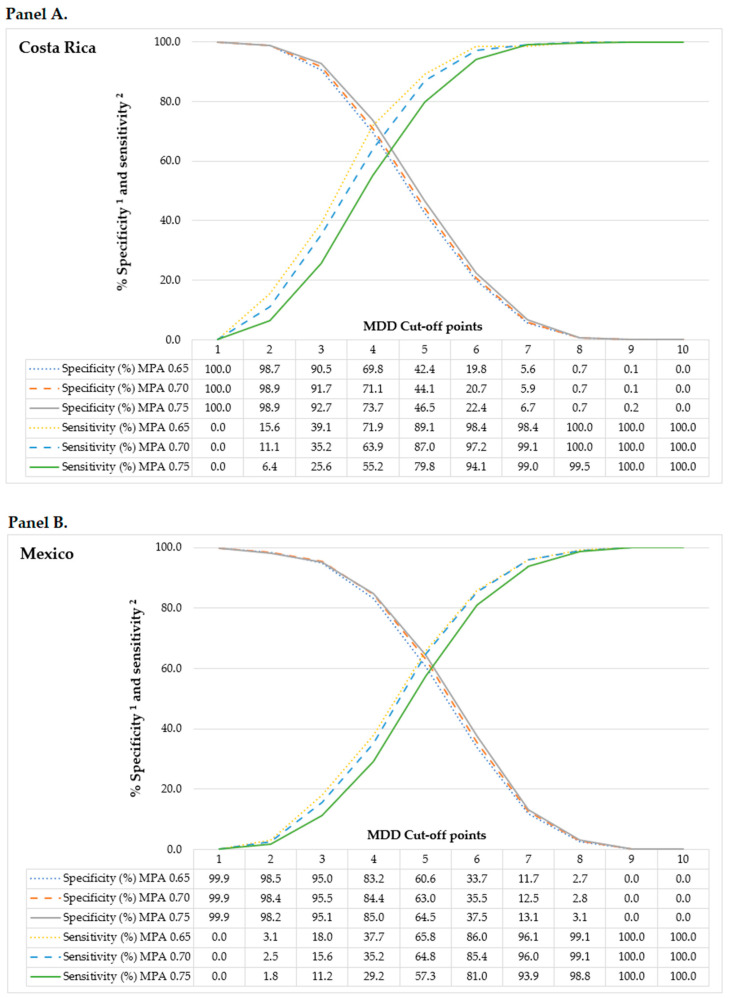
Percentage of specificity and sensitivity for diverse MPA values by 10 MDD cut-off points. (**Panel A**): CR adolescents, (**Panel B**): MX adolescents. ^1^ Specificity: identifies nutritionally appropriate diets as adequate. ^2^ Sensitivity: identifies nutritionally inappropriate diets as inadequate. MDD: Minimum Dietary Diversity; MPA: Mean Probability of Adequacy.

Receiver-operating characteristic (ROC) curves were utilized to determine the optimal MDD threshold for predicting MPA within each country’s sample. ROC curves are graphical representations of the relationship between the sensitivity and specificity of a diagnostic test. In this case, the gold standard test to determine nutritional adequacy is the MPA, while the MDD functions as a diagnostic accessory test [39]. Various MDD thresholds were examined against different definitions of a nutritionally adequate diet, revealing sensitivities and specificities ranging between 0.65 and 0.75 for MPA. Opting for an MPA of 0.70 as the threshold for a nutritionally adequate diet revealed area under the curve (AUC) values nearing 0.70, the highest values observed across both countries’ samples. Consequently, adhering to the FAO-established MPA cut-off point, an MPA of 0.70 was adopted for both countries [17]. Figure 1 demonstrates how sensitivity (probability of identifying nutritionally appropriate diets as adequate) and specificity (probability of identifying nutritionally inappropriate diets as inadequate) were marginally impacted by variations in the MPA cut-off points. More details about ROC curves methodology can be found elsewhere [39].

The most favorable MDD cut-off point is designed to maximize sensitivity in recognizing nutritionally inadequate diets while maintaining high specificity to correctly identify nutritionally adequate diets [21]. In the case of the CR sample, to predict an MPA ≥0.70, an MDD cut-off point equal to 4 resulted in 71.1% sensitivity, 63.9% specificity, and 70.2% correct classification rate with a Youden J-index value of 0.350; while to predict an MPA ≥0.70 for the MX sample, an MDD cut-off point equal to 5 resulted in 63.0% sensitivity, 64.8% specificity, and 63.5% correct classification rate with a Youden J-index value of 0.278. These MDD cut-off points resulted in values higher than 60% for sensitivity and specificity in both countries. Additionally, when assessing the Youden J-index, these chosen cut-off points displayed values closest to 1 in comparison to the values observed while analyzing alternative MDD cut-off points. This closer alignment with a Youden J-index value of 1 indicates a better balance between sensitivity and specificity, suggesting a more optimal discriminatory ability of these particular MDD cut-off points for the given samples in Costa Rica and Mexico.

**Figure 2:**
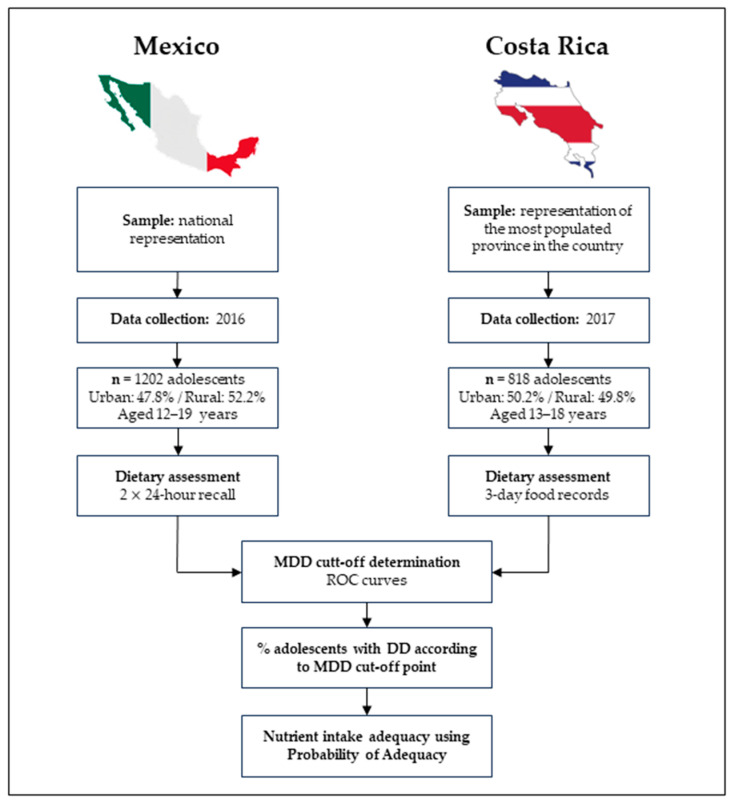
Methodological diagram showing characteristics of the Mexican and Costa Rican studies. MDD: minimum dietary diversity; ROC: receiver-operating characteristic; DD: diet diversity.

Figure 2 depicts a methodological diagram with the characteristics of the CR and MX studies.

### 2.8. Statistical Analyses

The general study population characteristics were presented as means, with their corresponding standard deviations (SD) for continuous variables and frequencies (%)used to describe categorical variables. For the mean MDD, a stratified analysis was conducted based on sex, area of residence, socioeconomic status (SES), and nutritional status. Differences between these groups were assessed using Student’s *t*-test, Wilcoxon test, or ANOVA, as deemed appropriate for the nature of the data. All statistical analyses were two-tailed, with the significance level set at *p*-values < 0.05.

For the dietary variables analysis, the consumption of food groups and nutrient intake was stratified by country, sex, and country area of residence and presented per 1000 kcal. PA for each nutrient and the MPA values were computed for each country sample. Differences between groups were assessed using the Wilcoxon test. The analyses were conducted using STATA software (version 14.1, 2015, College Station, TX, USA) [40] and IBM SPSS^®^ (version 27, IBM Corp., Armonk, NY, USA) [41].

## 3. Results

### 3.1. General Characteristics of the Study Participants by Country and Area of Residence

The average age of the CR sample was 15.0 years, with a standard deviation of 1.7 years (Table 1). The study sample was 63.6% female, 50.2% lived in urban areas, and 32.6% of the participants was classified as overweight or obese. There were no significant differences in age, sex, or overweight/obesity status between CR adolescents from urban and rural areas (*p* > 0.05). However, a higher proportion of rural adolescents were classified as having low socioeconomic status (SES) compared to urban adolescents (rural: 43.2% vs. urban: 21.2%; *p* < 0.0001).

The average age of the MX sample was 15.1 years, with a standard deviation of 2.3 years. The sample was nearly evenly split between male and female (49.0% female), with slightly less than half living in urban areas (47.8%). Similar to the CR sample, 32.5% of the MX sample was classified as overweight or obese. Adolescents from urban and rural areas in Mexico differed significantly in SES and nutritional status (*p* < 0.05), but not in age and sex. Similar to the CR sample, a lower proportion of urban MX adolescents were classified as having low SES compared to rural MX adolescents (rural: 46.3% vs. urban: 20.0%; *p* < 0.0001). Conversely, a higher proportion of urban MX adolescents was classified as overweight or obese compared to rural MX adolescents (rural: 32.5% vs. urban: 42.0%; *p* < 0.0001).

### 3.2. Minimum Dietary Diversity by the General Characteristics of the Study Participants

The mean MDD for the overall CR sample was 4.17 ± 1.43 points, and for the MX sample, the mean MDD was 4.68 ± 1.48 points out of the 10.0 possible maximum score (Table 2). Using the corresponding cut-off points described in Panel A and B of Figure 1, the percentage of adolescents with a diverse diet was significantly higher in Costa Rica than in Mexico (66.5% and 55.6%, respectively, *p* < 0.0001).

In the CR adolescents, those residing in rural areas exhibited a higher MDD (4.33 ± 1.43) compared to their urban counterparts (4.00 ± 1.42; *p* = 0.001). Notably, 72.0% of rural adolescents met the criterion for a diverse diet, surpassing the 61.1% observed among urban adolescents (*p* = 0.001). However, within the CR sample, no significant differences were identified when analyzing MDD based on sex, socioeconomic status (SES), or nutritional status. Conversely, in the MX sample, no significant differences emerged concerning the proportion of adolescents with diverse diets across sex, area of residence, SES, or nutritional status.

### 3.3. Consumption of Food Groups

By 1000 kcal, in Costa Rica, boys had a significantly higher consumption of starchy staples and pulses than girls; on the contrary, dietary intake of other fruits and other vegetables was significantly higher in girls compared to boys (Table 3). In Mexico, girls had a higher intake of other fruits, other vegetables, and other vitamin A-rich F&V than boys. There were no significant differences in the consumption of flesh foods, eggs, milk and milk products, nuts and seeds, and dark green leafy vegetables between boys and girls in both Costa Rica and Mexico.

Analysis of food consumption by area of residence showed that in Costa Rica and Mexico, adolescents living in rural areas had a significantly higher consumption of starchy staples and pulses per 1000 kcal than urban adolescents (Table 4). Additionally, CR but not MX rural adolescents had a significantly higher intake of eggs, other vegetables, and dark green leafy vegetables. In contrast, CR urban adolescents had a higher dietary intake of milk and milk products than rural ones. On the other hand, MX urban adolescents had a higher intake of flesh foods, milk and milk products, and other vitamin A-rich F&V than rural adolescents.

There were no differences in the consumption of nuts and seeds and other fruits between urban and rural CR and MX adolescents. Dietary intake of dark green leafy vegetables was similar between urban and rural MX adolescents; likewise, flesh foods consumption was similar between urban and rural CR adolescents.

**Figure 3:**
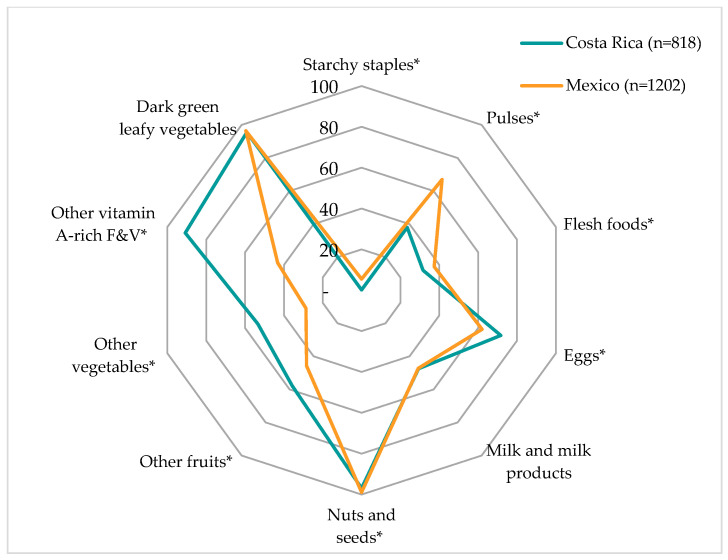
Percentage of Costa Rican and Mexican adolescents with consumption lower than 15 g for each of the ten food groups. F&V: fruits and vegetables. * Food groups with significant differences between countries (chi-square test, *p* < 0.05).

Figure 3 shows the proportion of CR and MX adolescents who consumed less than 15 g/d of each food group by country. Compared to MX adolescents, a higher proportion of CR adolescents consumed less than 15 g/d of eggs (71.6% vs. 62.0%; *p* < 0.0001), other fruits (57.8% vs. 45.8%; *p* < 0.0001), other vegetables (53.4% vs. 28.6%; *p* < 0.0001), and other vitamin A-rich F&V (90.8% vs. 43.2%; *p* < 0.0001). On the other hand, a higher proportion of MX adolescents than CR adolescents consumed less than 15 g/d of starchy staples (5.5% vs. 0.1%; *p* < 0.0001), pulses (67.0% vs. 38.0%; *p* < 0.0001), flesh foods (37.4% vs. 31.7%; *p* = 0.008), and nuts and seeds (98.9% vs. 96.9%; *p* = 0.001). There were no significant differences in milk and milk products or dark green leafy vegetables; however, it should be noted that close to 95% of adolescents in both countries consumed less than 15 g/d of dark green leafy vegetables.

**Figure 4:**
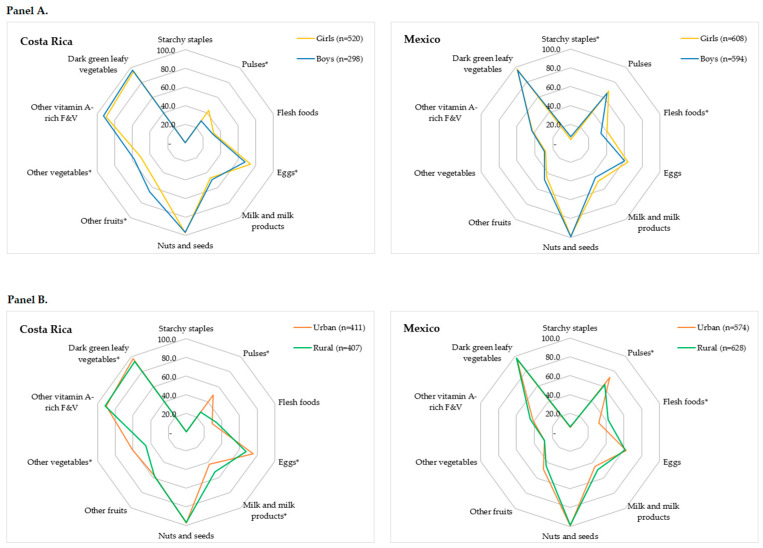
Percentage of adolescents whose consumption of the different food groups was less than 15 g. (**Panel A**): Data set by sex, (**Panel B**): data set by area of residence. F&V: fruits and vegetables. * Food groups with significant differences between categories of sex and area of residence (chi-square test, *p* < 0.05).

Figure 4 illustrates the distribution of adolescents who ingested less than 15 g/d of each food group across countries, categorized by sex (panel A) and area of residence (panel B). The percentage of Costa Rican adolescents consuming less than 15 g/d of pulses (43.1% vs. 29.2%; *p* < 0.0001) and eggs (74.0% vs. 67.4%; *p* = 0.044) was higher in girls; for other fruits (65.4% vs. 53.5%; *p* = 0.001) and other vegetables (58.1% vs. 50.8%; *p* = 0.044), the percentage was higher in boys. In Mexico, the percentage of adolescents consuming less than 15 g/d of flesh foods was higher in girls (40.6% vs. 34.0%; *p* = 0.018), and for starchy staples, it was higher in boys (6.9% vs. 4.1%; *p* = 0.034).

Regarding the area of residence, in Costa Rica, the percentage of consuming less than 15 g/d of pulses (49.4% vs. 26.5%; *p* < 0.0001), eggs (75.4% vs. 67.8%; *p* = 0.016), other vegetables (60.8% vs. 45.9%; *p* < 0.0001), and dark green leafy vegetables (97.3% vs. 93.9%; *p* = 0.016) was higher in rural areas; and for milk and milk products (52.3% vs. 42.6%; *p* = 0.016), it was higher in urban areas. In Mexico, the percentage of adolescents consuming less than 15 g/d of pulses (71.8% vs. 62.6%; *p* < 0.0001) was higher in urban areas, and for flesh foods (42.2% vs. 32.1%; *p* < 0.0001), it was higher in rural areas.

### 3.4. Nutrient Probability of Adequacy

**Figure 5:**
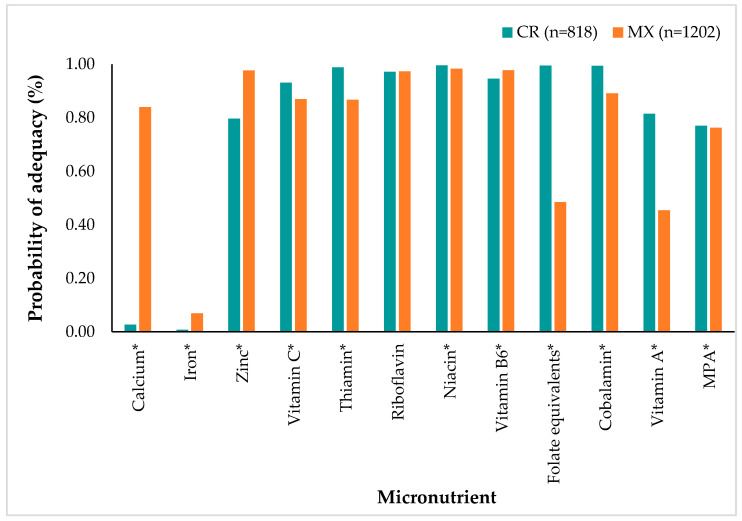
Probability of Adequacy (PA) for each nutrient and Mean Probability of Adequacy (MPA) by country. * Nutrients with significant differences between countries, determined using the Wilcoxon test (*p* < 0.05).

Figure 5 shows the probability of adequacy (PA) for each nutrient and the mean probability of adequacy (MPA) by country. CR adolescents had higher PA than MX participants for 6 of the 11 micronutrients assessed: vitamin C, thiamin, niacin, folate equivalents, cobalamin, and vitamin A. On the other hand, MX adolescents had significantly higher PA than CR adolescents for calcium, iron, zinc, and vitamin B6. There was no significant difference in the riboflavin PA between countries (*p* = 0.085). MPA was significantly higher in the CR group than in MX adolescents (CR: 0.77 vs. MX: 0.76; *p* = 0.014).

The calcium PA in CR adolescents was critically lower than in MXs (CR: 0.03 vs. MX: 0.84; *p* < 0.0001), while low PA was obtained for iron in both countries (CR: 0.01 vs. MX: 0.07; *p* < 0.0001). Also, the folate equivalents and vitamin A PA were notoriously low in MX adolescents, which was significantly lower than in CR (folate equivalents: CR: 0.99 vs. MX: 0.49; *p* < 0.0001; vitamin A: CR: 0.82 vs. MX: 0.45); *p* < 0.0001).

For additional information, Figure S1 shows a comparison between CR and MX adolescents by sex (panel A) and area of residence (panel B). Overall, when comparing countries using the same variables, the patterns in nutrient PA remain as shown in Figure 4. Also, for micronutrient PA, Tables S1 and S2 offer a comparison between sexes (Table S1) and area of residence (Table S2) by country.

## 4. Discussion

This study shows that the MDD cut-off point was lower for CR versus MX adolescents, suggesting that the consumption of at least four food groups contributes to CR adolescents having a sufficiently diverse diet to achieve adequate micronutrient intakes, while MX adolescents require the consumption of at least five food groups. The MDD cut-off point resulted from a detailed determination and analysis of sensitivity and specificity, which sought to balance these two aspects as a strategy to avoid the potential risk of overestimating the percentage of adolescents with insufficient micronutrient intakes. This balance was achieved at different MDD cut-off points for each country, which is consistent with previous evidence that recommends determining the cut-off point in different sociocultural contexts as there may be differences related to food cultures [5,20,21,22].

Unlike the findings in other studies [42,43,44,45], the CR diet was more diverse in rural than in urban areas. An explanation may be that rural adolescents have a higher consumption of starchy staples, pulses, eggs, other vegetables, and dark green leafy vegetables. The differences in the consumption of these food groups previously made it possible to determine that the odds of having a diverse diet are 62% higher in rural adolescents than in their urban counterparts [6].

In Mexico, there was no difference in dietary diversity between urban and rural adolescents, even though the poverty rate is higher in rural areas and the purchasing power of rural households is only half the average income of urban households [46]. However, urban adolescents have a higher consumption of flesh foods, milk and milk products, and other vitamin A-rich F&V (high-cost foods), which could contribute to equating the dietary diversity of urban areas with rural areas, where the consumption of starchy staples and pulses (low-cost foods) is higher.

Despite the limited socioeconomic situation of MX rural areas, the higher intake of the mentioned food groups may be a consequence of self-consumption such as corn and beans, which have been important in traditional subsistence agriculture and the main rural family life strategy [47,48,49].

In contrast to rural areas in Mexico, Costa Rica’s rural landscape has undergone a notable transformation in its productive structure. Here, the economic framework is no longer solely reliant on agricultural endeavors; there has been substantial growth in service-related activities that extend beyond conventional agriculture, such as banking services, education and health services, the manufacturing of fabric and leather goods, and ecological tourism services, among others [50]. Additionally, a distinct process of functional integration between rural and urban environments is evident, described as a form of counter-urbanization [50]. This phenomenon reflects a growing interconnection between these once-distinct settings. Such integration has been facilitated by the emergence and adoption of new communication technologies. Moreover, the rise in household income within rural areas contributes significantly to bridging the divide between urban and rural spheres. This socioeconomic progress plays a crucial role in narrowing the gap between previously disparate urban and rural contexts, fostering greater cohesion and interdependence. This becomes more relevant when considering that the characteristics of the rural areas included in this study are similar to those of the rest of the country.

Even though the overall MDD cut-off point for CR adolescents was lower (4.17 ± 1.43 out of 10 points) than the one for MX adolescents (4.68 ± 1.48), the PA for six micronutrients (vitamin C, thiamin, niacin, folate equivalents, cobalamin, and vitamin A) was significantly higher in CR adolescents. The notable difference in the PA of folate equivalents and vitamin A is particularly surprising. The low consumption of beans, fruits, and vegetables rich in folate equivalents, as well as foods rich in vitamin A, has previously been reported in MX and CR adolescents [51,52]. Therefore, the difference evidenced in the PA of folate equivalents could be a consequence of the robust CR fortification programs of wheat flour, corn flour, rice, and milk with folic acid and sugar for household consumption and milk with vitamin A, which are monitored yearly [53]. Although corn and wheat flours are also fortified with folic acid in Mexico—and in even greater quantities than in Costa Rica (2 mg/kg vs. 1.3 mg/kg of corn flour and 2 mg/kg vs. 1.8 mg/kg of wheat flour)—compliance is lower than in Costa Rica: 65% for corn flour and 87.5% for wheat flour [54] vs. 100% compliance for rice, wheat flour, and corn flour in Costa Rica [55]. As a public health strategy, better monitoring of folic acid fortification programs could contribute to improving the dietary intake of this micronutrient in MX adolescents and preventing the negative health outcomes associated with its deficiency. Vitamin A fortification could also be considered a public health strategy to improve the dietary intake of this micronutrient in the MX adolescent population.

Contrary to what was observed in the PA of folic acid and vitamin A, the PA of zinc was significantly higher in MX adolescents. This could have been explained by a higher consumption of beans, but it is still considerably low in Mexico [56]. The fortification of corn and wheat flours in Mexico with 40 mg of zinc/kg [54] may be the main reason. In Costa Rica, for instance, only rice is fortified with zinc and in smaller amounts (7.5 mg/kg) [55]. This result suggests that CR public health decision-makers should consider improving food fortification with zinc.

An enormous difference in calcium PA was observed between CR and MX adolescents (0.03 and 0.84, respectively), which may be explained by considering the main sources of calcium in the adolescent diet. In Costa Rica, milk and milk products provide 35% of the total intake for this micronutrient, and consumption is low among adolescents [57]. In Mexico, nixtamalized corn tortillas are an important source of calcium since, on average, it provides ~103 mg/100 g of corn tortilla [34]. Corn tortillas are high in calcium because they are processed by soaking boiled corn overnight in a lime solution containing calcium hydroxide, a process called nixtamalization [58]. The contribution from this food, together with milk, dairy products, and other traditional sources of calcium, significantly increases calcium intake in MX adolescents.

The calcium PA in both MX and CR adolescents is low but particularly worrying in the latter. However, it is important to consider that the adolescents’ EAR for calcium (1100 mg/d), proposed in 2011 [59] by the Institute of Medicine/National Academy of Medicine (IOM/NAM) of the United States, has been considered very high and difficult to achieve [60]; therefore, the recommended calcium dietary intake for the adolescent population must be evaluated by the MX and CR public health stakeholders in light of new proposals developed more recently by other international organizations such as the Nordic Council of Ministers [61].

A problem that adolescents from both countries share is the low PA of iron (0.01 in Costa Rica and 0.07 in Mexico). This could be a reflection of the trend toward lower bean consumption in both countries [6,56], which would provide significant amounts of nonheme iron [62]. In addition, the iron fortification programs for corn flour and wheat flour in Mexico and for corn flour, wheat flour, and milk in Costa Rica do not appear to be having the desired effect. One possible explanation may be the limited bioavailability of the fortificant (ferrous fumarate) used in the fortification of wheat flour in Mexico and Costa Rica and corn flour in Mexico because of the high concentration of iron bioavailability inhibitors in these foods [63]. This represents only one possible explanation; achieving true causality requires an exhaustive analysis of sociodemographic and dietary variables, as well as the biological key players involved in maintaining iron homeostasis. It may also be possible that fortified foods such as milk and corn flour are insufficiently consumed in Costa Rica, even though the fortificant (iron bisglycinate) is highly bioavailable [64].

The findings of this study should be interpreted within the context of some inherent limitations. Primarily, the sample of adolescents from Mexico is nationally representative, but the one from Costa Rica may not provide a complete representation of the entire country. Nevertheless, they accurately depict the province of San José, which is the most populated province in the country [65] and houses the largest proportion of the national adolescent population [25]. Secondly, different methods were used to assess diet intake: 24HR was used in Mexico versus 3FR in Costa Rica. Nevertheless, both methods are suitable for obtaining good nutrient intake estimates [66]. While both studies are not completely comparable, they do allow for an exploration of the landscape regarding the diversity and adequacy of micronutrient intake within an age group usually excluded from dietary assessments. The study also has strengths. First, the sample of adolescents is large and allows for the assessment of differences in dietary diversity and micronutrient adequacy among strata. Second, robust epidemiological tools were used to determine the MPA and MDD cut-off points, in accordance with FAO methodology.

## 5. Conclusions

The MDD cut-off point varied between adolescents in Costa Rica and Mexico, underscoring the non-uniform nature of MDD and its dependence on the distinct dietary patterns and food supply characteristics of each population group. The study proposes that including four food groups in the diet of CR adolescents and five in that of MX adolescents is adequate to fulfill their nutritional requirements for most micronutrients. This adequacy may stem from the inherent nutrient density of the included food groups or their function as carriers of various fortificants. The research highlights the importance of establishing MDD cut-off points tailored to each population group, emphasizing the pivotal role of dietary idiosyncrasy in this determination.

While the deficiency in the prevalence of most micronutrients was minimal, targeted interventions are essential to enhance the consumption of beans, fruits, and vegetables rich in vitamin A. This approach aims to improve the prevalence of vitamin A, folate equivalents, iron, and zinc. Furthermore, diligent monitoring of food fortification programs with folic acid in Mexico is imperative to mitigate the adverse effects associated with insufficient consumption of this micronutrient during embryogenesis, such as spina bifida, myelomeningocele, and anencephaly [67].

Public health decision-makers in both Costa Rica and Mexico should assess the effectiveness of fortifying various foods with iron, given the considerably low prevalence of iron adequacy in adolescents in both countries. Additionally, the discussion tables of public health stakeholders in Costa Rica and Mexico should address the issue of low prevalence of calcium adequacy, particularly in Costa Rica.

## Figures and Tables

**Table 1:** Characteristics of the Costa Rican and Mexican adolescents included in the study by area of residence.

Characteristics	Costa Rica				Mexico			
	Overall ^1^ (*n* = 818)	Area of Residence			Overall ^1^ (*n* = 1202)	Area of Residence		
		Urban (*n* = 411)	Rural (*n* = 407)	*p*-Value ^2^		Urban (*n* = 574)	Rural (*n* = 628)	*p*-Value ^2^
Age (y)	15.0 ± 1.7	14.9 ± 1.7	15.1 ± 1.7	0.208	15.1 ± 2.3	15.2 ± 2.3	15.0 ± 2.2	0.078
Sex								
Girls	520 (63.6)	259 (63.0) ^a^	261 (64.1) ^a^	0.741	594 (49.4)	300 (52.3) ^a^	308 (49.0) ^a^	0.265
Boys	298 (36.4)	152 (37.0) ^a^	146 (35.9) ^a^		608 (50.6)	274 (47.7) ^a^	320 (51.0) ^a^	
Socioeconomic status								
Low	263 (32.2)	87 (21.2) ^a^	179 (43.2) ^b^	<0.0001	406 (33.8)	115 (20.0) ^b^	291 (46.3) ^a^	<0.0001
Middle	325 (39.7)	158 (38.4) ^a^	167 (41.2) ^a^		433 (36.0)	195 (34.0) ^a^	238 (37.9) ^a^	
High	230 (28.1)	166 (40.4) ^a^	64 (15.7) ^b^		363 (30.2)	264 (46.0) ^b^	99 (15.8) ^a^	
Nutritional status								
Non-overweight	551 (67.4)	279 (67.9) ^a^	272 (66.8) ^a^	0.748	757 (63.0)	333 (58.0) ^b^	424 (67.5) ^a^	<0.0001
Overweight/obesity	267 (32.6)	132 (32.1) ^a^	135 (33.2) ^a^		445 (37.0)	241 (42.0) ^b^	204 (32.5) ^a^	
Energy intake (kcal)	1873 ± 763	1852 ± 769	1896 ± 757	0.459	1913 ± 898	1939 ± 949	1888 ± 849	0.707

^1^ Values are means ± SD or frequencies (%) unless otherwise indicated. ^a,b^ Labeled frequencies in a row without a common letter differ from expected values (*p* < 0.05). ^2^ *p*-values < 0.05 are statistically significant and were determined using the Wilcoxon test, or the chi-square test with Bonferroni post hoc test when required. The *p*-values compare means of age or proportions in categories of sex, socioeconomic status, and nutritional status between categories of area of residence (urban/rural).

**Table 2:** Percentage of Costa Rican and Mexican adolescents with a diverse diet.

Characteristic	Costa Rica (*n* = 818)			CR Participants with a Diverse Diet (MDD ≥ 4)		Mexico (*n* = 1202)			MX Participants with a Diverse Diet (MDD ≥ 5 )	
	Mean	SD	*p*-Value ^1^	*n* (%)	*p*-Value ^1^	Mean	SD	*p*-Value ^1^	*n* (%)	*p*-Value ^1^
Overall	4.17	1.43		544 (66.5)		4.68	1.48		668 (55.6)	<0.0001 ^2^
Sex										
Girls	4.18	1.43	0.679	349 (67.1)	0.624	4.62	1.38	0.177	337 (56.7)	0.424
Boys	4.14	1.44		195 (65.4)		4.74	1.58		337 (54.4)	
Area of residence										
Urban	4.00	1.42	0.001	251 (61.1)	0.001	4.70	1.51	0.632	323 (56.3)	0.642
Rural	4.33	1.43		293 (72.0)		4.66	1.46		345 (54.9)	
Socioeconomic status										
Low	4.28	1.44	0.303	183 (69.6)	0.420	4.50 ^a^	1.37	0.010	211 (52.0)	0.194
Middle	4.11	1.39		213 (65.5)		4.79 ^b^	1.50		247 (57.0)	
High	4.11	1.48		148 (64.4)		4.74 ^a,b^	1.56		210 (57.9)	
Nutritional status										
Non-overweight	4.15	1.43	0.680	367 (66.6)	0.929	4.70	1.48	0.545	427 (56.4)	0.449
Overweight/obesity	4.19	1.43		177 (66.3)		4.65	1.49		241 (54.2)	

^1^ *p*-values < 0.05 are statistically significant and were determined using the chi-square, Student’s t, Wilcoxon, or ANOVA tests. ^2^ Chi-square test for the percentage of adolescents with a diverse diet between countries. ^a,b^ Labeled means in a column without a common letter are statistically different.

**Table 3:** Food groups consumed among Costa Rican and Mexican adolescents by sex.

Food Group (g/d/1000 kcal) ^1^	Costa Rica (*n* = 818)			Mexico (*n* = 1202)		
	Girls (*n* = 520)	Boys (*n* = 298)	*p*-Value ^2^	Girls (*n* = 608)	Boys (*n* = 594)	*p*-Value ^2^
Starchy staples	204.2 ± 84.0	214.2 ± 81.6	0.037	133.2 ± 88.5	133.5 ± 89.8	0.957
Pulses	41.2 ± 52.8	58.8 ± 60.4	<0.0001	10.7 ± 19.6	12.2 ± 22.9	0.515
Flesh foods	47.0 ± 47.5	45.1 ± 43.6	1.000	42.8 ± 56.7	45.3 ± 54.9	0.125
Eggs	9.5 ± 21.1	11.5 ± 21.6	0.063	19.9 ± 34.7	22.1 ± 41.3	0.319
Milk and milk products	77.1 ± 115.9	72.7 ± 107.6	0.475	76.7 ± 114.4	82.7 ± 116.1	0.222
Nuts and seeds	0.9 ± 6.8	0.7 ± 4.8	0.807	0.4 ± 4.0	0.2 ± 1.8	0.148
Other fruits	45.3 ± 83.2	34.1 ± 89.4	0.001	67.1 ± 102.5	58.1 ± 104.7	0.022
Other vegetables	27.6 ± 45.7	22.6 ± 50.5	0.021	46.2 ± 79.5	38.7 ± 67.2	0.041
Other vitamin A-rich F&V	7.7 ± 31.9	5.5 ± 36.4	0.086	30.5 ± 91.8	27.7 ± 76.1	0.046
Dark green leafy vegetables	3.3 ± 20.3	2.2 ± 15.3	0.270	1.1 ± 7.5	1.5 ± 9.9	0.650

^1^ Values are means ± SD by 1000 kcal. ^2^ *p*-values < 0.05 are statistically significant and were determined using the Wilcoxon test. F&V: fruits and vegetables.

**Table 4:** Consumption of food groups of adolescents by country and area of residence.

Food Group (g/d/1000 kcal) ^1^	Costa Rica (*n* = 818)			Mexico (*n* = 1202)		
	Urban (*n* = 411)	Rural (*n* = 407)	*p*-Value ^2^	Urban (*n* = 574)	Rural (*n* = 628)	*p*-Value ^2^
Starchy staples	199.1 ± 90.0	216.7 ± 74.9	<0.0001	121.3 ± 84.9	144.4 ± 91.4	<0.0001
Pulses	41.4 ± 58.5	54.0 ± 53.3	<0.0001	9.8 ± 21.4	12.9 ± 21.0	<0.0001
Flesh foods	48.8 ± 46.9	43.7 ± 45.2	0.111	47.3 ± 56.0	41.0 ± 55.6	0.002
Eggs	9.6 ± 22.4	10.8 ± 20.2	0.039	20.9 ± 40.1	21.1 ± 36.2	0.913
Milk and milk products	84.1 ± 122.5	66.9 ± 101.7	0.006	89.1 ± 120.2	71.0 ± 110.0	0.005
Nuts and seeds	0.8 ± 5.5	0.9 ± 6.7	0.461	0.3 ± 2.5	0.3 ± 3.6	0.871
Other fruits	42.5 ± 95.2	40.0 ± 74.9	0.865	64.3 ± 109.4	61.1 ± 98.1	0.767
Other vegetables	21.9 ± 51.3	29.7 ± 43.2	<0.0001	43.4 ± 72.0	41.7 ± 75.4	0.164
Other vitamin A-rich F&V	8.4 ± 40.2	5.4 ± 25.2	0.405	36.8 ± 116.6	22.1 ± 33.4	0.008
Dark green leafy vegetables	1.4 ± 10.7	4.5 ± 24.0	0.015	1.1 ± 5.2	1.5 ± 11.1	0.072

^1^ Values are means ± SD by 1000 kcal. ^2^ *p*-values < 0.05 are statistically significant and were determined using the Wilcoxon test. F&V: fruits and vegetables.

## Data Availability

The data presented in this study are available on request from the corresponding author. The data are not publicly available due to privacy and ethical restrictions.

## Supplementary Materials

The following supporting information can be downloaded at: https://www.mdpi.com/article/10.3390/children11010064/s1, Figure S1. Probability of Adequacy (PA) for each nutrient and Mean Probability of Adequacy (MPA) by country, according to sex (panel A) and residence area (panel B). * Nutrients with significant differences between countries, determined using the Wilcoxon test (*p* < 0.05). Table S1. Probability of Adequacy (PA) for Costa Rican and Mexican adolescents, according to sex. Table S2. Probability of Adequacy (PA) for Costa Rican and Mexican adolescents, according to residence area.
