# Supplementary material for: Exploring Differences in Dietary Diversity and Micronutrient Adequacy between Costa Rican and Mexican Adolescents
Source: Children (Basel). 2024 Jan 3;11(1):64. doi: 10.3390/children11010064 (PMC10814227; doi:10.3390/children11010064)
Supplement: Supplementary file 1 [file children-11-00064-s001.zip › children-2759084-supplementary.pdf]

children

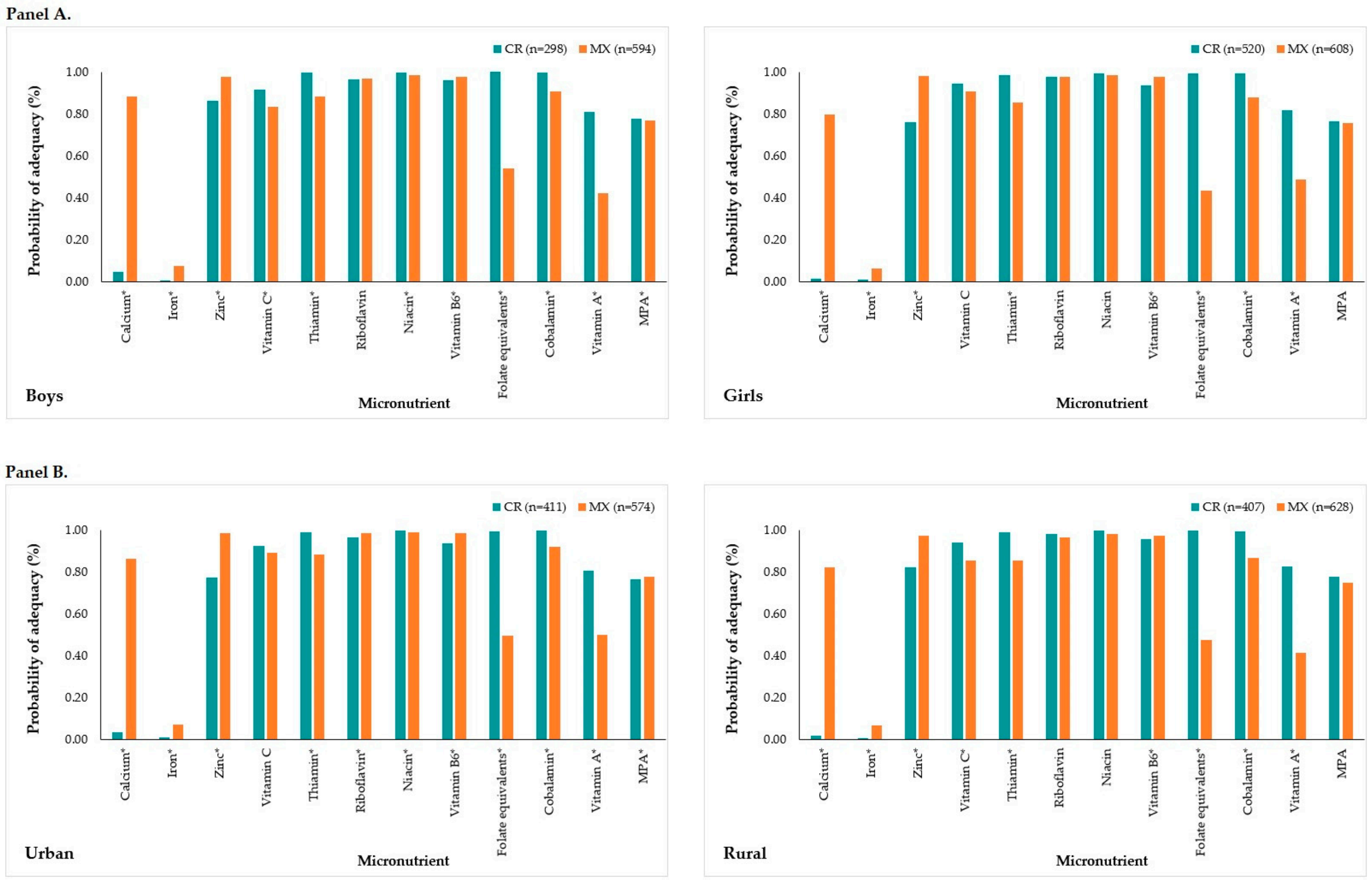

Figure S1. Probability of Adequacy (PA) for each nutrient and Mean Probability of Adequacy (MPA) by country, according to sex (panel A) and residence area (panel B). * Nutrients with significant differences between countries, determined using the Wilcoxon test ($p < 0.05$).

Table S1. Probability of Adequacy (PA) for Costa Rican and Mexican adolescents, according to sex.

Nutrient [1]	Costa Rica (n = 818)					Mexico (n = 1202)				
	Girls (n = 520)		Boys (n = 298)			Girls (n = 608)		Boys (n = 594)		
	Mean	SD	Mean	*SD*	*p*-value [1]	Mean	SD	Mean	*SD*	*p*-value [1]
Calcium	0.01	0.09	0.05	0.18	<0.0001	0.80	0.35	0.88	0.28	<0.0001
Iron	0.01	0.03	0.01	0.05	0.326	0.06	0.14	0.08	0.15	0.278
Zinc	0.76	0.30	0.86	0.25	<0.0001	0.98	0.09	0.97	0.12	0.693
Vitamin C	0.94	0.20	0.91	0.25	0.307	0.91	0.28	0.83	0.35	<0.0001
Thiamin	0.98	0.08	1.00	0.03	<0.0001	0.85	0.31	0.88	0.29	0.011
Riboflavin	0.98	0.12	0.96	0.16	0.628	0.98	0.13	0.97	0.14	0.149
Niacin	0.99	0.04	1.00	0.02	0.001	0.98	0.08	0.98	0.09	0.524
Vitamin B6	0.94	0.19	0.96	0.14	0.001	0.98	0.12	0.98	0.13	0.005
Folate equivalents	0.99	0.07	1.00	0.00	<0.0001	0.43	0.46	0.54	0.47	<0.0001
Cobalamin	0.99	0.06	1.00	0.03	0.002	0.88	0.29	0.91	0.26	0.011
Vitamin A	0.82	0.28	0.81	0.27	0.582	0.49	0.41	0.42	0.41	0.001
MPA	0.77	0.09	0.78	0.08	0.005	0.76	0.15	0.77	0.15	0.066

[1] *p*-values < 0.05 are statistically significant and were determined using the Wilcoxon test. MPA: Mean Probability of Adequacy.

Table S2. Probability of Adequacy (PA) for Costa Rican and Mexican adolescents, according to residence area.

**Nutrient** [1]	**Costa Rica (*n* = 818)**					**Mexico (*n* = 1202)**				
	**Urban (*n* = 411)**		**Rural (*n* = 407)**			**Urban (*n* = 574)**		**Rural (*n* = 628)**		
	**Mean**	**SD**	**Mean**	**SD**	***p*-value** [1]	**Mean**	**SD**	**Mean**	**SD**	***p*-value** [1]
Calcium	0.03	0.14	0.02	0.11	0.084	0.86	0.30	0.82	0.33	0.002
Iron	0.01	0.03	0.01	0.04	0.143	0.07	0.15	0.07	0.14	0.207
Zinc	0.77	0.30	0.82	0.28	0.014	0.98	0.08	0.97	0.13	0.416
Vitamin C	0.92	0.22	0.94	0.21	0.006	0.89	0.29	0.85	0.34	0.051
Thiamin	0.99	0.07	0.99	0.07	0.190	0.88	0.28	0.85	0.31	0.100
Riboflavin	0.96	0.15	0.98	0.11	0.129	0.98	0.10	0.97	0.16	<0.0001
Niacin	1.00	0.03	1.00	0.03	0.566	0.99	0.07	0.98	0.10	0.565
Vitamin B6	0.94	0.18	0.96	0.16	0.004	0.98	0.11	0.97	0.14	0.772
Folate equivalents	0.99	0.05	0.99	0.06	0.706	0.50	0.46	0.47	0.47	0.597
Cobalamin	1.00	0.04	0.99	0.06	0.384	0.92	0.24	0.87	0.30	0.001
Vitamin A	0.80	0.28	0.83	0.27	0.421	0.50	0.41	0.41	0.41	<0.0001
MPA	0.76	0.09	0.77	0.08	0.034	0.78	0.16	0.75	0.16	0.002

[1] *p*-values < 0.05 are statistically significant and were determined using the Wilcoxon test. MPA: Mean Probability of Adequacy.
